# Case report: Right atrium-inferior vena cava bypass in a patient with unusual cardiac cystic echinococcosis

**DOI:** 10.3389/fcvm.2022.1001073

**Published:** 2022-11-03

**Authors:** Lulu Liu, Binggang Wu, Mei Li, Yingqiang Guo

**Affiliations:** Department of Cardiovascular Surgery, West China Hospital, Sichuan University, Chengdu, China

**Keywords:** hydatid disease, cardiac echinococcosis, right atrium mass, inferior vena cava obstruction, vascular bypass graft

## Abstract

Cardiovascular hydatid disease is caused by parasitic infection of *Echinococcus granulosus*, which could be asymptomatic or life-threatening depending on lesion site, granuloma size, and disease progression. Diagnosis and treatment of cardiac echinococcosis should be under comprehensive consideration. In this case, we reported a successful right atrium-inferior vena cava bypass surgery in a 31-year-old female with unresectable right atrial echinococcosis and inferior vena cava obstruction.

## Introduction

Hydatid disease is caused by *Echinococcus* species, which remains to be a common and severe public health problem in husbandry areas. Cystic echinococcosis is responsible for 95% of human hydatid cases. Humans are accidental hosts and are usually infected by ingesting parasitic ova (1). Cardiac involvement is uncommon for hydatid disease, which is usually found in the liver and lungs. Most of the patients are asymptomatic and the discovery of cardiac echinococcosis is often incidental. However, cardiac hydatid disease might lead to severe complications, including severe allergic reaction resulting from intracardiac rupture of hydatid cyst and sudden death caused by pulmonary embolism or acute valvular obstruction (2). Although there is no official guideline for the treatment of cardiac hydatid disease, surgical R0 resection associated with albendazole-based chemotherapy is usually preferred (3). For the patients whose cardiac lesion could not achieve R0 resection, the perioperative management and clinical outcome remain to be a tough problem. We reported a case in which palliative vascular bypass surgery was performed on a patient with unresectable right-atrium echinococcosis.

## Case presentation

### Case description

**FIGURE 1:**
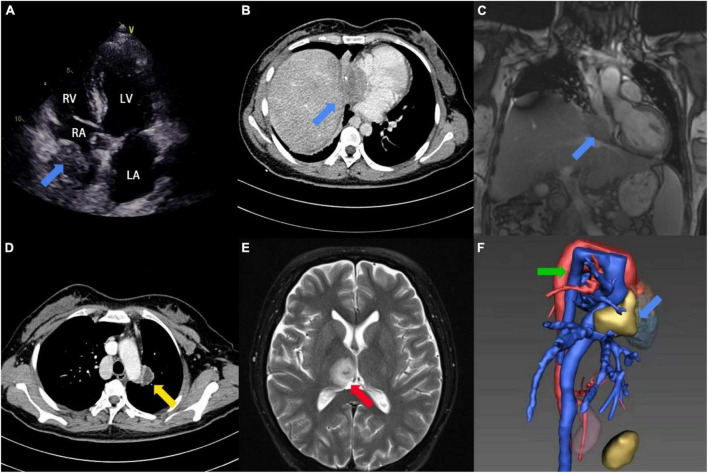
Image findings. **(A)** Preoperative 2D-TTE four-chamber view showed an irregular mass (blue arrow) within the right atrium. **(B)** Preoperative CTA showed a mass with slight calcification (blue arrow) within the right atrium and IVC. **(C)** Preoperative MRI showed a hypointense mass (blue arrow) at the junction of the right atrium and IVC on coronary-reconstruction view. **(D)** Preoperative CTA showed a lesion with slight enhancement in the left lung (yellow arrow). **(E)** Preoperative MRI showed an oval hypertense lesion (red arrow) within the brain on T2-weighted image. **(F)** 3D-reconstruction view of preoperative CTA showed the cardiac mass (blue arrow) and enlarged azygos vein (green arrow).

A 31-year-old female patient was admitted to the hospital with repeated hemoptysis and dyspnea on exertion for 3 years. In addition, a fever reaching 39°C and watery diarrhea had occurred 4 days before admission. Long-term residence history in an endemic area of echinococcosis was confirmed on admission. On physical examination, the patient presented with persistent tachycardia of 102 beats per minute. Besides, the cardiac murmur was not found during auscultation of the heart. Peripheral blood test demonstrated concurrent moderate anemia with the hemoglobin (Hb) of 85 g/L and eosinophilia with the eosinophil percentage (EO%) of 10.6%. The hepatic function test showed elevation in total bilirubin (TBIL, 32.1 μmol/L) and direct bilirubin (DBIL, 16.4 μmol/L), while alanine transaminase (ALT) and aspartate transaminase (AST) remained normal. Furthermore, detection of the echinococcosis granulosus IgG was positive. Preoperative transthoracic echocardiography (TTE) revealed a 24 mm × 50 mm immobile mass extending from the inferior vena cava (IVC) into the right atrium. There was no clear boundary between the epicardium and the mass (Figure 1A). Computed tomography angiogram (CTA) and magnetic resonance imaging (MRI) confirmed the mass, while multiple lesions were also found in the left lung and the brain (Figures 1B–E and Supplementary Video 1). In addition, the azygos vein dilated significantly as a compensatory sign of IVC obstruction (Figure 1F). Transbronchoscopic lung biopsy demonstrated that the pathological diagnosis of the left lung lesion was granulomatous inflammation resulting from echinococcosis infection.

### Diagnostic assessment

**FIGURE 2:**
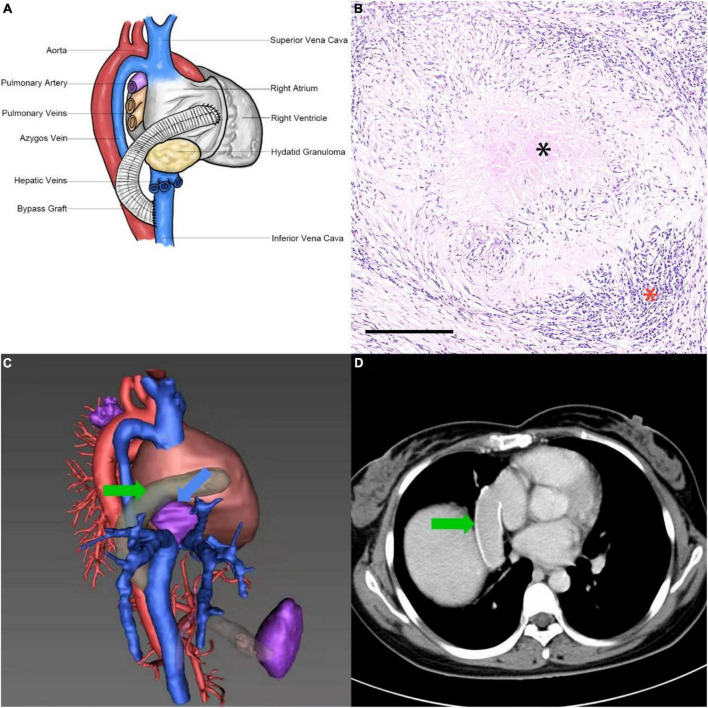
Surgical technique and postoperative findings. **(A)** Illustration of the right atrium-IVC bypass. **(B)** Postoperative pathological analysis showed the existence of cysticercosis (black asterisk), aggregation of eosinophils and proliferation of epithelioid cells (red asterisk). H/E 10×. The black bar indicates 250 μm. **(C)** 3D-reconstruction view of postoperative CTA on 5-year follow-up showed the cardiac mass without enlargement (blue arrow) and vascular graft (green arrow). **(D)** Postoperative CTA on 5-year follow-up showed the vascular graft remained patent (green arrow).

After 4-week therapy with albendazole (400 mg, three times per day), cardiac surgery was performed to relieve obstruction of IVC. The pericardium was opened following a median sternotomy and the extracorporeal circulation was established by cannulation of the right femoral artery, right femoral vein and superior vena cava (SVC). Then antegrade cardioplegic perfusion was performed and a right atriotomy was applied to expose the mass. A piece of 24 mm × 50 mm gray hard tissue was found within the right atrium, which invaded outward to form tight adhesions with the pericardium, right phrenic nerve, right lower lung, and diaphragm. The mass also invaded downward and resulted in severe obstruction of the IVC. The intracavitary portion of the mass and part of the right atrium wall were removed for test and the diagnosis of intraoperative frozen section examination was granulomatous inflammation. Complete resection of the mass was evaluated to be impossible after rigorous discussion among cardiovascular surgeons, cardiologists, and infectiologists, therefore the IVC was fully exposed with the assistance of the right-sided thoracotomy and a right atrium-IVC bypass was performed. One side of a 20 mm polytetrafluoroethylene vascular graft (W. L. Gore & Associates, Inc., USA) was connected to the right atrial appendage by end-to-side anastomosis and another side was connected to the IVC with end-to-side anastomosis, which was just lower than the level of the second porta hepatis (Figure 2A). Bovine pericardial patch was used to repair the defect of right atrium. The patient went through an uncomplicated postoperative period in the intensive care unit (ICU) and proceeded to continuous anti-parasitic therapy with albendazole (400 mg, twice per day) for maintenance treatment after the operation. In addition, anti-coagulation therapy was not administrated. The postoperative pathological analysis confirmed the diagnosis of echinococcosis granulosus (Figure 2B). The patient was discharged from the hospital 30 days after the surgery and remained in good condition of cardiac function and hepatic function at the time of the 5-year follow-up. The TTE and CTA revealed that the vascular graft remained patent and there was no enlargement in the mass (Figures 2C,D). Moreover, no thrombo-hemorrhage event, anaphylactic reaction, new lesion or other complication was found during the 5-year follow-up.

### Table timeline

**Table:** 

Day 0
• A 31-year-old female was admitted into the department of cardiovascular surgery due to repeated hemoptysis and dyspnea on exertion.
• Physical examination revealed persistent tachycardia.
Laboratory test evidenced moderate anemia, eosinophilia, bilirubinemia, and positive echinococcosis granulosus IgG.
Day 1
• Transthoracic echocardiogram revealed an immobile mass in the right atrium.
Day 2
• Computed tomography angiogram and magnetic resonance imaging confirmed the inferior vena cava obstruction and multiple lesions in the left lung and the brain.
Day 7
• Transbronchoscopic lung biopsy demonstrated the left pulmonary lesion was hydatid granuloma.
Day 8
• Albendazole was prescribed prior to the cardiovascular surgery.
Week 4
• Right atrium-inferior vena cava bypass surgery was performed.
• Histological confirmation of cardiac hydatid granuloma.
Year 5
• Follow-up Doppler echocardiography and computed tomography angiogram demonstrated patent vascular graft and no mass progression.
• Patient was asymptomatic. Cardiac function and hepatic function returned to normal.

## Discussion

Hydatid disease is classified as a parasitic infection resulting from the larvae of *Echinococcus granulosus* and most of the patients had a residence history in pastoral areas. The most common source of transmission is the domestic dog and the major route of infection transmission is intaking food and water containing the ova of the parasite (4). Cardiac involvement of hydatidosis is rare in humans, which accounts for 0.2–2% of all cases, while most common lesions occur in the liver (50–70%) and lung (20–30%). Among the heart involvement cases, the left ventricle is the dominant susceptible area (55–60%), followed by the right ventricle (10–20%), interventricular septum (5–9%), left atrium (8%), pulmonary arteries (7–8%), pericardium (5%), and right atrium (3–4%) (5). Thus, involvement of the right atrium is extremely rare in human cases.

Depending on the growth rate, mass volume and lesion site, cardiac hydatid disease could be asymptomatic or life-threatening. Most of the patients have no symptoms due to the slow growth of the granuloma. However, about 10% of the patients might have variable complications, including pericarditis, pericardial effusion, anaphylactic reaction, systemic embolism, pulmonary embolism, and myocardial infarction (6). In this case, the hydatidosis invaded the right atrium, leading to severe obstruction of the IVC and symptoms like Budd-Chiari syndrome, which was uncommon among cardiac-involved cases.

Diagnosis of hydatid disease relies on imaging examination and pathological analysis. The TTE, CTA, and MRI are sensitive in detecting lesion sites and involving the extent of cardiac hydatidosis. Multiple lesions in the liver, lung and other parts of the body might assist in the diagnosis of cardiac hydatidosis (7). Confirmation of cardiac echinococcosis depends on postoperative pathological examination, of which the major signs include the presence of cysticercosis, aggregation of eosinophils and proliferation of epithelioid cells. Seropositive echinococcosis granulosus IgG in enzyme-linked immunosorbent assay might also help diagnose cardiac echinococcosis.

Surgical treatment and medicinal therapy were the two major components of the management of hydatid disease (8). Surgical removal of echinococcosis granuloma with cardiopulmonary bypass under cardioplegic arrest is the optimal choice for cardiac hydatid disease. However, hydatid granuloma tends to grow subendocardially and form tight adhesion with adjacent structures (7). In our case, the cardiac mass invaded the right atrium, IVC, pericardium, right lower lung and diaphragm, which resulted in obstruction of IVC and could not be resected completely. Under this circumstance, the right atrium-IVC bypass surgery might be a feasible option to restore venous circulation. Chemotherapy with albendazole is equally important in treating cardiac echinococcosis. It is recommended to use prophylactic chemotherapy a few weeks prior to the cardiac operation and continued after the surgery to inhibit the growth of hydatid granuloma and reduce the risks of recurrence (9). Chemotherapy with albendazole plus praziquantel could be used as adjunctive treatment to surgery (1).

## Conclusion

Right atrium echinococcosis is extremely rare and could possibly cause IVC obstruction. Chemotherapy associated with right atrium-IVC bypass surgery might be a feasible treatment when hydatid granuloma could not be removed completely.

## Data availability statement

The original contributions presented in this study are included in the article/Supplementary material, further inquiries can be directed to the corresponding author.

## Ethics statement

The studies involving human participants were reviewed and approved by the West China Hospital Ethics Committees and Institutional Review Board, Sichuan, China. The patients/participants provided their written informed consent to participate in this study. Written informed consent was obtained from the individual(s) for the publication of any potentially identifiable images or data included in this article.

## Author contributions

LL and BW: conception and design, data analysis, and interpretation. YG: administrative support. LL, BW, and ML: collection and assembly of data. All authors wrote the manuscript and approved the final manuscript.

## Supplementary material

Supplementary Video 1 Preoperative magnetic resonance imaging showed a 24 mm × 50 mm mass existing at the junction of the right atrium and IVC.
